# LC–ESI–MS/MS-based molecular networking, antioxidant, anti-glioma activity and molecular docking studies of *Clematis graveolens*

**DOI:** 10.1186/s13007-024-01221-3

**Published:** 2024-07-25

**Authors:** Zubair Ahmed, Muhammad Ikram, Ishaq Khan, Kashif Bashir, Abdul Jabbar Shah, Zahid Hussain, Taous Khan

**Affiliations:** 1 Department of Pharmacy, COMSATS University Islamabad, Abbottabad campus, 22060 Abbottabad, KP Pakistan; 2 Department of Molecular Biology and Genetics, Institute of Basic Medical Sciences, Khyber Medical University, 25000, Peshawar, Pakistan; 3 Texas A&M Health Science Centre, Joe H. Reynolds Medical Build 159, College Station, 77843 Texas USA; 4 Department of Pharmaceutical Sciences, Pak Austria Fachhochschule: Institute of Applied Sciences and Technology, 22600, Haripur, Pakistan; 5 Department of Chemistry, COMSATS University Islamabad, Abbottabad campus, 22060 Abbottabad, KP Pakistan

**Keywords:** Antioxidant activity, Anti-glioma activity, Molecular networking, Molecular docking studies, LC–MS/MS profiling

## Abstract

*Clematis graveolens* Lindl.*,* an indigenous climbing plant found in the Himalayan areas, is used by local communities for the treatment of neck tumors. The objective of this work is to examine the comprehensive metabolomic profile, antioxidant capability, in vitro and in silico anti-glioma effects on U-87 human glioma cell lines of the crude extract and fractions from *C. graveolens*. Liquid chromatography coupled with mass spectroscopy (LC–MS/MS) was used to establish detailed metabolite profiling of *C. graveolens*. The assessment of cell cytotoxicity was conducted using MTT cell viability assay on U-87 and BHK-21. Through molecular docking studies, the mode of inhibition and binding interaction between identified compounds and target proteins were also determined to evaluate the in vitro results. The use of LC–MS/MS-based global natural products social (GNPS) molecular networking analysis resulted in the identification of 27 compounds. The crude extract, ethyl acetate fraction, and chloroform fraction exhibited significant inhibitory activity against the U-87 cell lines, with IC_50_ values of 112.0, 138.1, and 142.7 µg/mL, respectively. The ethyl acetate fraction exhibited significant inhibitory concentration for 2,2’-azino-bis (3-ethylbenzothiazoline-6-sulfonic acid) (ABTS) activity, 2,2-diphenyl-1-picrylhydrazyl (DPPH) activity and the metal chelation activity with IC_50_ value of 39.50 µg/mL, 32.27 µg/mL, and 53.46 µg/mL, respectively. The crude extract showed maximum total phenolic, and total flavonoid concentration measuring 338.7 µg GAE/mg, and 177.04 µg QE/mg, respectively. The findings of this study indicate that *C. graveolens* consists of a diverse range of active phytoconstituents that possess antioxidant and anti-glioma properties.

## Introduction

Gliomas are the most frequently reported primary malignant brain tumors. They are distinguished by a high recrudescence rate, high cases of mortality, short survival times, and ineffectual treatment strategies. Approximately 26.6% of all brain tumors are gliomas [1]. The post-diagnosis survival rate of glioma patients (5.5%) is 5 years and even with multimodal treatment, which includes surgery, radiotherapy, and chemotherapy, the overall median survival is still abysmal at about 14.5–16.6 months [1]. The most dangerous type of this illness is glioblastoma, a stage IV glioma that makes up 54% of all gliomas and 15% of all primary brain tumors [2]. Treatment-associated glioma prognosis is still difficult since surgical excision is closely tied to the tumor's proximity to vital brain structures [2]. Furthermore, radiotherapy and chemotherapy have been futile [3]. When these tumors recur, they are usually resistant to further treatment with the same drugs [4]. Glioma patients' longevity and quality of life can be improved by developing novel treatments or therapeutic formulations that are both safe and economical [5]. Consequently, there seems to be a constant demand for new anticancer drugs that are both effective and affordable [6].

To treat various diseases, a vast variety of natural remedies have been employed for decades, with a focus on plants [7]. Plant secondary metabolites are present in the natural environment and have been identified as a promising novel reservoir of chemotherapeutic agents [8]. Alkaloids, saponins, terpenes, taxanes, and flavonoids have been supported by substantial data about their potential anticancer effects [9, 10]. Phenolic compounds have garnered significant attention in the scientific community as a highly researched category of bioactive chemicals, primarily due to their antioxidant properties and potential health benefits [11]. Based on a comprehensive analysis spanning the period from 1930 to 2014, it was shown that among the 246 anticancer medications that received approval from the Food and Drug Administration (FDA), a substantial majority of 177 drugs were either natural products or derived from such sources [12]. It is believed that these entities possess a higher degree of physiological compatibility, indicating a greater degree of co-evolution with their respective target environments and a reduced likelihood of posing harm to regular cells. Increasing interest has been observed in the use of natural substances for the treatment of cancer. Based on data provided by the National Cancer Institute, a significant proportion of the currently active clinical studies for anti-cancer medications use compounds derived from natural sources [13].

Numerous active substances derived from natural sources are under investigation for their potential therapeutic use in the treatment of glioma. Epigallocatechin-3-gallate (EGCG) is a naturally occurring chemical that is present in green tea. Previous pre-clinical investigations have shown that EGCG may serve as a promising adjunctive therapeutic approach for glioma when used in addition to conventional therapies including surgery and radiation therapy [14]. A clinical trial (NCT01712542) performed on individuals diagnosed with preoperative glioblastoma showed that the use of curcumin along with radiation treatment effectively managed prolonged tumor development, while also exhibiting a favourable safety profile and high tolerability [15]. Irinotecan, a compound derived from camptothecin, is a naturally occurring alkaloid obtained from the plant species *Camptotheca acuminata*, indigenous to China. The combination of irinotecan, temozolomide, and radiation therapy is now under clinical studies for the treatment of anaplastic astrocytoma, glioblastoma, and oligodendroglioma [16]. The pharmacophore of sunitinib, an oxindole derivative, was first obtained from *Uncaria tomentosa*. Sunitinib is in phase II/III clinical studies for the therapeutic management of recurrent glioblastoma [17].

*Clematis graveolens* Lindl, often referred to as Chinjal-walla or Total in Pakistan which belongs to the family Ranunculaceae [18]. This plant has yellow blossoms and is found in the Himalayan areas of Afghanistan, Pakistan, Northwest India, and Western Nepal, specifically at altitudes of 900–3000 m [19]. It is cultivated through seeds and is sometimes grown as an ornamental plant. It causes blistering or scorching of skin, it is used in neck tumors and sore throat, haemorrhage, wounds healing, and as an antiseptic [18]. The leaves tincture prepared in spirit is used for the treatment of tumors of the neck [19]. Gas chromatography–mass spectrometry was used to establish the chemical constitution of the fixed oils and essential oils of *C. graveolens* [20]. However, there is limited research data available on the components of crude extract and their antioxidant and anti-glioma properties. Hence, the primary objective of this study was to comprehensively explore the chemical profile of *C. graveolens* using LC–MS/MS-based global natural product social molecular networking (GNPS). A quantitative analysis of phytochemicals was conducted to determine the total phenolic and flavonoid contents. In a similar vein, antioxidant experiments and in vitro cytotoxicity evaluations on human glioma cells were also performed. The phytochemical profile and pharmacological potentials of *C. graveolens* have been investigated for the first time, based on current understanding from the literature.

## Materials and methods

### Reagents

The following chemicals were acquired from Sigma Fine Chemicals (Uppsala, Sweden): Gallic acid (GA), ascorbic acid, phenol reagent, Follin Ciocalteu’s reagent, 1,1-diphenyl-2-picrylhydrazyl (DPPH), iron II chloride, trisodium citrate, and quercetin was obtained from Alfa Aesar, Ward Hill, MA, USA. High-performance liquid chromatography (HPLC) grade solvents, such as acetonitrile and methanol, were procured from Thermo Fisher Scientific, Waltham, MA, USA. Water for HPLC was obtained from Milli-Q Water Purification System, Millipore, Bedford, MA, USA. The formic acid used in this study was obtained from Acros Organics, Morris Plains, NJ, USA. All additional reagents used in the investigation were of analytical quality and were procured from reliable suppliers.

### Plant material

*C. graveolens* (whole plant) was collected in June 2018, from Swat, Khyber Pakhtunkhwa, Pakistan. The plant material was authenticated by a plant taxonomist, Dr. Zahid Ullah, Assistant Professor, Centre of Plant Sciences, University of Swat. The plant specimen with voucher number (SWAT002219) was deposited in the University herbarium for future record.

### Processing of plant material

To get rid of dust and other impurities, the collected plant material was rinsed with tap water. Plant material was allowed to dry naturally in the shade for two weeks followed by pulverization into fine powder in a home blender, which was then kept at room temperature in sealed containers until use.

### Extraction and fractionation

The powdered plant material (10 kg) was extracted with 80% methanol (20  L) initially for 21 days followed by 7, and 3 days, respectively at ambient temperature. The extracted plant material was concentrated at 40 °C under reduced pressure using a rotavapor (BUCHI Labortechnik AG, Flawil, Switzerland) to yield crude methanolic extract (750 g). Muslin cloth and Whatman 42 filter paper were used in the filtering process. The fractions were prepared by a solvent–solvent extraction process from crude methanol extract, following increasing order of polarity. The crude extract of 700 g was suspended in distilled water and fractionated with *n*-hexane, chloroform, ethyl acetate, and *n*-butanol, respectively [21].

### LC–ESI–MS/MS analysis

Crude extract of *C. graveolens* and its fractions were dissolved in respective HPLC grade solvents (1 mg/ml) and filtered using membrane filter (0.45 µm), vortexed, and sonicated. All the samples were refrigerated until further use. Each sample, 100 μL was transferred to the HPLC autosampler vials. The LC–MS/MS analysis was performed using the Dionex Ultimate 3000 UHPLC connected to the Orbitrap Velos Pro mass spectrometer (Thermo Fisher Scientific in Bremen, Germany). The analysis was performed in negative ion mode at a scan rate of 5 Hz. The stationary phase consisted of a C-18 reverse-phase column (UPLC Luna Omega) with dimensions of 150 mm in length, 2.1 mm in internal diameter, and a particle size of 1.6 μm. The mobile phases were composed of water (A) and acetonitrile (B) that were acidified using 0.1% formic acid. The gradient profile exhibited the following characteristics: an initial concentration of 5% (B) for a duration of 0.5 min, a transition from 5 to 95% (B) over a period of 0.5–6.5 min, a consistent concentration of 95% (B) from 6.5 to 8.5 min, and a subsequent transition from 95 to 5% (B) spanning 8.5–10 min. The temperature of the column was maintained at 30 °C. The flow rate of the mobile phase was maintained at 0.3 mL/min, while the injection volume of each sample was 10 μL [22]. The spectra were acquired using the full scan mode within the *m/z* range of 110–2000 amu. The collision-induced dissociation energy of the system under consideration was measured to be 35 V, while the resolution was determined to be 30,000 FWHM (full width and half maximum). The process of acquiring and interpreting the data was conducted via Thermo XCalibur program.

### Molecular networking

The molecular networking analysis of the extract of *C. graveolens* and its fractions based on polarity was conducted using the GNPS platform, which may be accessed online at https://gnps.ucsd.edu/ProteoSAFe/static/gnps-splash.jsp. The LC–MS/MS data was converted into “mzML” files that are compatible with GNPS using Proteowizard version 3.02.30260 MS Convert software. Subsequently, the files were transferred to the GNPS platform with the WinSCP program. The spectral networks' output findings were imported and visualized using Cytoscape 3.8.2. The sophisticated MolnetEnhancer tool was used to determine the primary categories of phytochemicals present in the extract and fractions of *C. graveolens*. The outcomes derived from the molecular networking analysis conducted using GNPS were collated with the outcomes derived from manual annotation and a thorough examination of existing literature [22].

### Determination of total phenolic contents (TPC)

TPC of the crude extract and fractions were determined using the Folin–Ciocalteu technique, as previously described [23], with minor modifications. The individual extract/fraction or gallic acid was dissolved in methanol at a concentration 1 mg/mL to prepare the stock solution. The 50 µL of each sample was introduced onto a 96-well microtiter plate after sonication for 10 min. Subsequently, 50 µL of 0.5 N Folin–Ciocalteu reagent was put in with the aforementioned samples, followed by an incubation period of 5 min. After incubation, 100 µL of sodium carbonate solution with a concentration of 20% was gently mixed with the samples and allowed to further incubate for 40 min at ambient temperature, while being covered from light. Subsequently, the measurement of absorbance was conducted at a wavelength of 750 nm using an IRMECO UV–Vis spectrophotometer (U2020, Germany). The quantification of the overall phenolic content was carried out using the calibration curve technique, with gallic acid serving as the standard.

### Determination of total flavonoid contents (TFC)

In order to ascertain the overall TFC levels in the crude extract and fractions, the aluminium chloride technique was used with minor adjustments, as mentioned earlier [21]. The samples were prepared by diluting 100 µg of extract/quercetin with 400 µL of methanol and the sonicated for 10 min. Subsequently, 100 µL of a 10% AlCl_3_ solution and 100 µL of a 1 M CH_3_COOK solution were added. The sample solution was left in a protected light-free container for 30 min. The absorbance of the sample solution was measured at 415 nm using an IRMECO UV–Vis spectrophotometer (U2020, Germany) against a blank. The quantification of the overall flavonoid content was carried out using the calibration curve, with quercetin serving as the standard.

### Antioxidant assays

To assess the antioxidant potential of *C. graveolens* crude extract and fractions, different protocols were followed as discussed below. For this purpose, the plant extract/fractions and ascorbic acid (standard) were dissolved in methanol. A standard curve was developed using successive dilutions spanning a concentration range of 25–75 µg/ml. The IC_50_ values were calculated from the graph plot of log of concentrations against percent inhibition by using linear equation:

$$y=mx+c$$

The experimental procedures included conducting tests and analyses in triplicate, after which the results were averaged. The % scavenging was calculated using the following equation:

$$\% Scavanging=\left[\frac{Absorbance\, of\, Control-Absorbance\, of\, Sample}{Absorbance\, of\, control} \right]\times 100$$

#### DPPH radical scavenging assay

The DPPH free radical scavenging activity of the crude extract and fractions was conducted using the technique outlined earlier [24], with minor adjustments. The radical’s stock solution was prepared by dissolving 24 mg of DPPH in 100 mL of methanol and afterward stored in a refrigerator for future use. In a test tube, a 3 mL solution of DPPH was mixed with 100 μL of each sample. The absorbance was measured at a wavelength of 517 nm using IRMECO UV–Vis spectrophotometer (U2020, Germany).

#### Ferrous metal ions chelating activity

The chelating activity of ferrous ions in the crude extract and fractions was assessed using the methodology described in the literature [25]. Subsequently, these diluted samples were introduced into a solution containing 0.05 ml of 2 mM FeCl_2_. Ferrozine solution of 0.2 ml (5 mM) was added to the mixture solution to initiate the reaction, which was followed by rapid agitation and allowed to stand at room temperature for 10 min. Following the attainment of equilibrium, the absorbance of the solution was quantified at a wavelength of 562 nm using an IRMECO UV–Vis spectrophotometer (U2020, Germany).

#### ABTS assay

The stock solution of ABTS was prepared by mixing 7 mM of ABTS reagent with 8.75 mM of potassium persulfate. This mixture was then incubated in a dark environment at room temperature for 16 h. Prior to use, the ABTS stock solution was further diluted to achieve a resulting concentration of 104.14 µM. Subsequently, a volume of 20 µL of each sample was added and mixed with 280 µL of a 104.14 µM ABTS solution in a 96-well plate. Following vertexing, the plate was incubated in darkness for 6 min. Finally, the absorbance was recorded at a wavelength of 734 nm using a microplate reader (Chem plate reader CR-201, China) [26].

#### Anti-glioma activity

Human glioma cell lines (U-87) and human non-cancerous kidney cell lines (BHK-21) acquired from (ATCC, Rockville, MD, USA) were used to perform in vitro anti-glioma activity and toxicity studies in normal cells, respectively. The culture cells were incubated at a temperature of 37 ℃ in Dulbecco's Modified Eagle Medium (DMEM) that was supplemented with 10% fetal bovine serum (FBS) and a combination of streptomycin and penicillin (100 µg/mL each). This incubation was carried out in a humidified environment containing 5% carbon dioxide (CO_2_). A total of 1 × 10^5^ cells per well were placed in 96-well plates and allowed to incubate for 24 h. Following this incubation period, the cells were subjected to specified doses of crude extract and fractions of *C. graveolens* for 48 h. Following the incubation period, the media was substituted with 100 µL of new media that included MTT at a concentration of 5 mg/ml. This mixture was then incubated at a temperature of 37 ℃ for 4 h. Dimethyl sulfoxide (DMSO) (100%) solution was used to dissolve the intracellular insoluble formazan salts. Subsequently, the absorbance of the solution was recorded at a wavelength of 492 nm using a microplate reader (Chem Plate Reader CR-201, China). The obtained absorbance values were then utilized to compute the percentage viability of the samples, as described previously [27]. DMSO (1%) solution was used as the negative control in the experiment. The percentage of inhibition was determined by using the following formula:

$$\% Viability=\left[\frac{Absorbance\, of\, Sample}{Absorbance\, of\, negative\, control} \right]\times 100$$

## Molecular docking studies

Glioma-related genes were acquired from the online databases such as GeneCards and DisGeNET databases. The prediction of the inhibitory potential was determined via docking studies against targeted proteins to investigate the binding interaction with the active site. X-ray crystal structure of protein having PDB IDs: 6S9W [28], 3AY5 [29], 7AEM [30], 4XVR [31], 4BQG [32], 6RFP [33], 6GES [34], 7JWE [35], and 6D3O [36] retrieved from Protein Data Bank was used as a starting structure for docking. The structures of synthesized compounds were drawn by using Chem Office 3D (2015) and their energies were optimized using Gaussian 09 employing the B3LYP density functional theory method with 6-311G basis set [37]. To prepare the protein model for docking, MGL tools were employed which involved the addition of Polar hydrogen and Kollman charges, repairing of missing residues of the active site [38]. After the protein preparation compound library was prepared and saved in the desire format a grid box was added according to the attributes (X, Y and Z) of the protein to facilitate the molecular docking via AutoDock Vina. The Lamarckian algorithm generated 50 poses for each confirmation and the top ranked pose was selected for further analysis. A Discovery visualizer (DSV) was used to determine the 2D and 3D models of the top ranked poses and binding interaction.

## Statistical analysis

The study of IC_50_ values of the results obtained was reported as the mean ± standard deviation (n = 3). The statistical analysis was conducted using GraphPad Prism 8.0.2. A statistical analysis was conducted to compare the mean IC_50_ values obtained for cancer cell lines. This analysis used a one-way ANOVA multiple comparisons test, where significance levels of *p* < 0.1 and *p* ≤ 0.001 were deemed to indicate statistical significance and strong statistical significance, respectively.

## Results and discussion

### LC–ESI–MS/MS-based molecular networking

*C. graveolens* crude extract and fractions were analysed using LC–MS/MS to create an in-depth phytochemical profile to examine the chemical diversity of the plant. Through the liquid chromatography-tandem mass spectrometry analysis, different metabolites were observed tentatively in both the crude extract and fractions. The annotated metabolites, together with their corresponding *m/z* values in negative ion mode, retention time, and exact mass values. The crude extract showed the presence of different phytoconstituents as shown in Table 1.

**Table 1:** Details of phytochemicals tentatively identified in *C. graveolens* using LC–ESI–MS/MS based molecular based networking analysis

S/No	Compound name	RT (min)	[M-H]^−^*m/z*	MS^2^ fragmentation ions	Molecular formula	Exact mass
Crude Extract of *C. graveolens*						
1	Schaftoside	3.73	563.23	443.10 (100), 473.11, 353.06, 383.08	C_26_H_28_O_14_	564.147
2	methoxy-myricetin-3-*O*-hexosyl-7-*O*-deoxyhexosyl (1–2) deoxyhexoside	3.83	787.17	623.16 (100), 315.01, 608.13, 331.04	C_34_H_42_O_21_	786.221
3	5-hydroxy-3-(5-hydroxy-2,4-dimethoxyphenyl)-6-methoxy-7-[3,4,5-trihydroxy-6-(hydroxymethyl) oxan-2-yl] oxychromen-4-one (Iridin)	3.90	521.21	358.81 (100), 461.20, 388.92	C_24_H_26_O_13_	522.137
4	Rutin	3.92	609.14	298.05 (100), 299.05, 607.17, 300.06	C_27_H_30_O_16_	610.153
5	Vitexin	3.95	431.11	310.61 (100), 268.44, 340.70	C_21_H_20_O_10_	432.105
6	Isoquercetin	4.00	463.08	300.54 (100), 299.61, 301.44	C_21_H_20_O_12_	464.095
7	Sissotrin	4.21	447.09	283.04 (100), 284.07, 445.12	C_22_H_22_O_10_	446.121
8	Kaempferol-3-*O*-rutinoside	4.22	593.15	284.49 (100), 283.62, 256.45	C_27_H_30_O_15_	594.158
9	Prunin	4.42	435.42	274.5 (100), 392.02, 418.03	C_21_H_22_O_10_	434.121
10	Rhoifolin	4.90	579.13	269.45 (100), 270.43, 432.98, 311.60	C_27_H_30_O_14_	578.163
11	Elatoside E	5.80	881.54	603.59 (100), 749.89, 750.90, 471.27	C_46_H_74_O_16_	882.497
12	Ceanothic acid	6.07	485.27	416.81(100), 450.18, 424.86,	C_30_H_46_O_5_	486.334
13	Isorhamnetin-3,7-di*-O*-glucoside	6.33	638.32	476.34 (100), 278.75, 518.40	C_28_H_32_O_17_	640.163
14	(2-aminoethoxy) [(2R)-2-[(9Z)-hexadec-9-enoyloxy]-3-hydroxypropoxy] phosphinic acid	6.67	452.27	255.24 (100), 196.05, 214.05	C_21_H_42_NO_7_P	451.269
15	N-hexadecanoyl-sn-glycero-3-phosphoethanolamine	6.67	453.28	255.24 (100), 255.85, 196.05	C_21_H_43_NO_7_P	453.285
16	Ginsenoside Rb3	9.49	1121.64	1077.57 (100), 1078.62, 1076.6	C_53_H_90_O_22_	1078.592
17	Ginsenoside Rb2	9.53	1121.65	1077.42 (100), 1078.31, 1076.69, 1091.36	C_53_H_90_O_22_	1078.592
*n*-Hexane fraction						
18	Homoarginine	2.27	190.92	146.07 (100), 161.54, 145.18	C_7_H_16_N_4_O_2_	188.127
1*	Schaftoside	3.73	563.23	443.10 (100), 473.11, 353.06, 383.08	C_26_H_28_O_14_	564.147
4*	Rutin	3.92	609.14	298.05 (100), 299.05, 607.17, 300.06	C_27_H_30_O_16_	610.153
5*	Vitexin	3.95	431.11	310.61 (100), 268.44, 340.70	C_21_H_20_O_10_	432.105
6*	Isoquercetin	4.00	463.08	300.54 (100), 299.61, 301.44	C_21_H_20_O_12_	464.095
7*	Sissotrin	4.21	447.09	283.04 (100), 284.07, 445.12	C_22_H_22_O_10_	446.121
8*	Kaempferol-3-*O*-rutinoside	4.22	593.15	284.49 (100), 283.62, 256.45	C_27_H_30_O_15_	594.158
10*	Rhoifolin	4.90	579.13	269.45 (100), 270.43, 432.98, 311.60	C_27_H_30_O_14_	578.163
19	9,12,13-trihydroxyoctadeca-10,15-dienoic acid	4.97	327.21	228.80 (100), 171.15, 290.88	C_18_H_32_O_5_	328.224
11*	Elatoside E	5.80	881.54	603.59 (100), 749.89, 750.90, 471.27	C_46_H_74_O_16_	882.497
12*	Ceanothic acid	6.07	485.27	416.81(100), 450.18, 424.86,	C_30_H_46_O_5_	486.334
13*	Isorhamnetin-3,7-di-*O*-glucoside	6.33	638.32	476.34 (100), 278.75, 518.40	C_28_H_32_O_17_	640.163
14*	(2-aminoethoxy) [(2R)-2-[(9Z)-hexadec-9-enoyloxy]-3-hydroxypropoxy] phosphinic acid	6.67	452.27	255.24 (100), 196.05, 214.05	C_21_H_42_NO_7_P	451.269
15*	N-hexadecanoyl-sn-glycero-3-phosphoethanolamine	6.67	453.28	255.24 (100), 255.85, 196.05	C_21_H_43_NO_7_P	453.285
20	Phosphatidylethanolamine lyso	7.47	451.17	254.60 (100), 420.31, 362.15	C_21_H_44_NO_7_P	453.285
Chloroform fraction						
18*	Homoarginine	2.27	190.92	146.07 (100), 161.54, 145.18	C_7_H_16_N_4_O_2_	188.127
3*	5-hydroxy-3-(5-hydroxy-2,4-dimethoxyphenyl)-6-methoxy-7-[3,4,5-trihydroxy-6-(hydroxymethyl) oxan-2-yl] oxychromen-4-one (Iridin)	3.90	521.21	358.81 (100), 461.20, 388.92	C_24_H_26_O_13_	522.137
4**	Rutin	3.92	609.14	298.05 (100), 299.05, 607.17, 300.06	C_27_H_30_O_16_	610.153
21	Methyl robustone	4.07	377.16	346.80 (100), 344.74,345.76	C_22_H_18_O_6_	378.110
19*	9,12,13-trihydroxyoctadeca-10,15-dienoic acid	4.97	327.21	228.80 (100), 171.15, 290.88	C_18_H_32_O_5_	328.224
11**	Elatoside E	5.80	881.54	603.59 (100), 749.89, 750.90, 471.27	C_46_H_74_O_16_	882.497
14**	(2-aminoethoxy) [(2R)-2-[(9Z)-hexadec-9-enoyloxy]-3-hydroxypropoxy] phosphinic acid	6.67	452.27	255.24 (100), 196.05, 214.05	C_21_H_42_NO_7_P	451.269
15**	N-hexadecanoyl-sn-glycero-3-phosphoethanolamine	6.67	453.28	255.24 (100), 255.85, 196.05	C_21_H_43_NO_7_P	453.285
16*	Ginsenoside Rb3	9.49	1121.64	1077.57 (100), 1078.62, 1076.6	C_53_H_90_O_22_	1078.592
17**	Ginsenoside Rb2	9.53	1121.65	1077.42 (100), 1078.31, 1076.69, 1091.36	C_53_H_90_O_22_	1078.592
Ethyl acetate fraction						
22	(2-[2,6-dimethylphenyl)-methoxy acetylamino] propionic acid	3.35	266.13	160.11 (100), 148.11, 192.14	C_14_H_18_NO_4_	265.131
3**	5-hydroxy-3-(5-hydroxy-2,4-dimethoxyphenyl)-6-methoxy-7-[3,4,5-trihydroxy-6-(hydroxymethyl) oxan-2-yl] oxychromen-4-one (Iridin)	3.90	521.21	358.81 (100), 461.20, 388.92	C_24_H_26_O_13_	522.137
4***	Rutin	3.92	609.14	298.05 (100), 299.05, 607.17, 300.06	C_27_H_30_O_16_	610.153
5**	Vitexin	3.95	431.11	310.61 (100), 268.44, 340.70	C_21_H_20_O_10_	432.105
6**	Isoquercetin	4.00	463.08	300.54 (100), 299.61, 301.44	C_21_H_20_O_12_	464.095
7**	Sissotrin	4.21	447.09	283.04 (100), 284.07, 445.12	C_22_H_22_O_10_	446.121
8**	Kaempferol-3-*O*-rutinoside	4.22	593.15	284.49 (100), 283.62, 256.45	C_27_H_30_O_15_	594.158
9*	Prunin	4.42	435.42	274.5 (100), 392.02, 418.03	C_21_H_22_O_10_	434.121
10**	Rhoifolin	4.90	579.13	269.45 (100), 270.43, 432.98, 311.60	C_27_H_30_O_14_	578.163
23	Isovitexin	5.36	431.22	315.68 (100), 368.39, 310.61	C_21_H_20_O_10_	432.105
24	6-[5,7-dihydroxy-2-(4-hydroxyphenyl)-4-oxochromen-6-yl]-5,7-dihydroxy-2-(4-hydroxyphenyl) chromen-4-one	5.42	537.08	375.05 (100), 417.06, 376.05,	C_30_H_18_O_10_	538.089
11***	Elatoside E	5.80	881.54	603.59 (100), 749.89, 750.90, 471.27	C_46_H_74_O_16_	882.497
*n*-Butanol fraction						
1**	Schaftoside	3.73	563.23	443.10 (100), 473.11, 353.06, 383.08	C_26_H_28_O_14_	564.147
2*	methoxy-myricetin-3-*O*-hexosyl-7-*O*-deoxyhexosyl (1–2) deoxyhexoside	3.83	787.17	623.16 (100), 315.01, 608.13, 331.04	C_34_H_42_O_21_	786.221
3***	5-hydroxy-3-(5-hydroxy-2,4-dimethoxyphenyl)-6-methoxy-7-[3,4,5-trihydroxy-6-(hydroxymethyl) oxan-2-yl] oxychromen-4-one (Iridin)	3.90	521.21	358.81 (100), 461.20, 388.92	C_24_H_26_O_13_	522.137
4****	Rutin	3.92	609.14	298.05 (100), 299.05, 607.17, 300.06	C_27_H_30_O_16_	610.153
5***	Vitexin	3.95	431.11	310.61 (100), 268.44, 340.70	C_21_H_20_O_10_	432.105
6***	Isoquercetin	4.00	463.08	300.54 (100), 299.61, 301.44	C_21_H_20_O_12_	464.095
8***	Kaempferol-3-*O*-rutinoside	4.22	593.15	284.49 (100), 283.62, 256.45	C_27_H_30_O_15_	594.158
9**	Prunin	4.42	435.42	274.5 (100), 392.02, 418.03	C_21_H_22_O_10_	434.121
25	Neohesperidin dihydrochalcone	4.44	611.32	449.17 (100), 431.14, 286.65	C_28_H_36_O_15_	612.205
26	Dihydroxy-2',5',5',8a'-tetramethyl-6-oxo-3,3',4',4a',5',6,6',7,7',8,8',8a'-dodecahydro-2'H-spiro[furo[2,3-e] isoindole-2,1'-naphthalen]-7'-yl acetate	5.17	489.26	442.22 (100), 383.20, 400.21	C_25_H_33_NO_6_	443.533
27	Melezitose	5.44	529.13	365.11 (100), 347.09, 527.16	C_18_H_32_O_16_	504.169
Aqueous fraction						
28	[3-[(9Z,12Z)-octadeca-9,12-dienoyl] oxy-2-[(9Z,12Z,15Z)-octadeca-9,12,15-trienoyl] oxypropyl] [(5R)-2,3,4,5,6-pentahydroxycyclohexyl] phosphate	7.49	855.51	575.4 (100), 577.4, 412.99, 278.59	C_45_H_76_O_13_P	855.502

^**^, ***, **** represent compounds observed in crude extract and also observed in one or two subsequent fractions, respectively

**Fig. 1:**
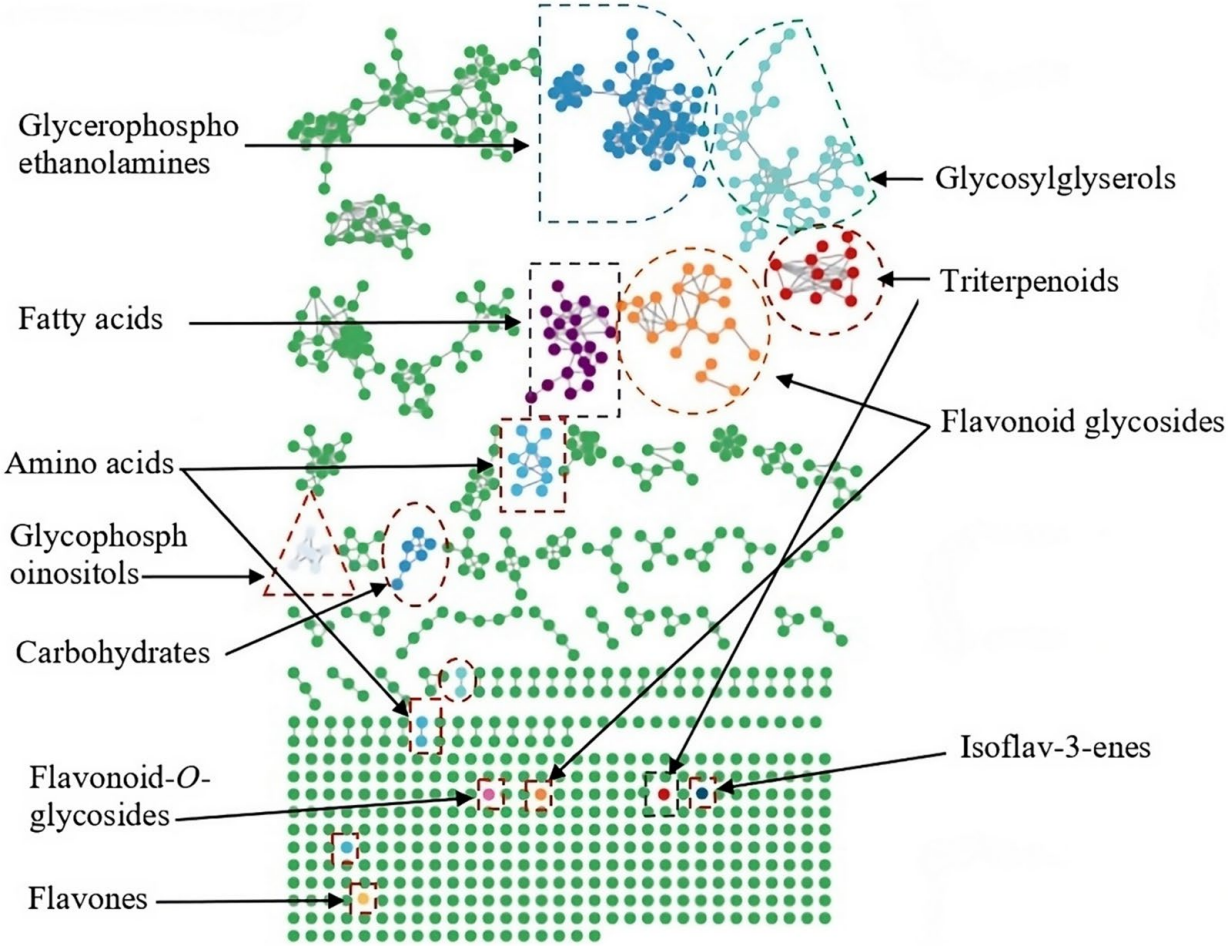
Annotation of cluster nodes according to metabolites classes in *C. graveolens* crude extract and fractions through molecular networking

GNPS platform is a community-led knowledge space in which natural products (plant, microbes, marine etc.) data can be shared, analysed, and annotated by researchers worldwide. It enables a cycle of annotation in which users curate data, identify products dereplication and share reference spectral libraries [39].The GNPS data analysis process was used to conduct molecular networking, using the spectral clustering technique (GNPS, http://gnps.ucsd.edu). Based on the similarity of metabolites fragmentation patterns, a detailed molecular network was annotated through converted MS/MS data. A total of 714 nodes were annotated and then categorized based on their compound classes, as seen in Fig. 1.

**Fig. 2:**
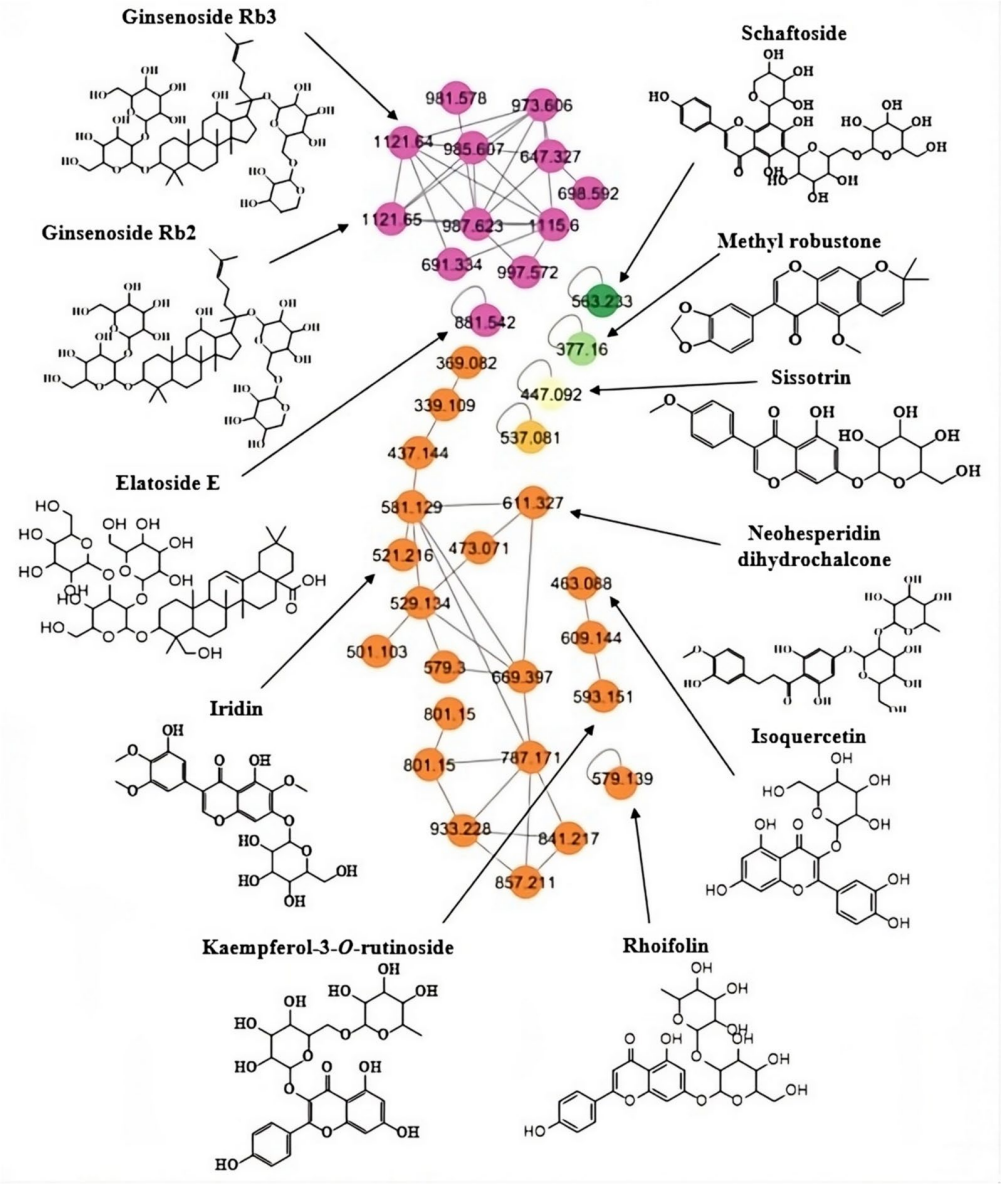
Structures of metabolites and their parent mass identified through GNPS molecular networking in *C. graveolens*

The major molecular networks flavonoid-*O*-glycosides included isoquercetin, rhoifolin, kaempferol-3-*O*-rutinoside, iridin, and neohesperidin dihydrochalcone clustered together. Sissotrin was annotated as isoflavonoid-*O*-glycoside while schaftoside was identified as flavonoid-di-*C*-glycoside. Ginsenoside Rb2, Rb3, and elatoside E were identified as major triterpenoid saponins. The outcomes of the GNPS analysis were carefully compared with the findings obtained via manual annotation, taking into account factors such as m/z, MS–MS fragmentation, and molecular formula. To ensure the presence of various phytometabolites, selected nodes were briefly dereplicated as seen in Fig. 2.

Isoquercetin was observed with the depicted mass of *m/z* 463.08 Da, lost its glucose unit and underwent quercetin fragmentation ions at *m/z* 300.54 Da, 299.61 Da, and 301.44 Da [40]. Isoquercetin inhibited cancer growth in many cell lines via interacting with the Wnt signalling system, mixed-lineage protein kinase 3, mitogen-activated protein kinase, apoptotic pathways, and proinflammatory protein signalling [41]. Isorhamnetin-3,7-di*-O*-glucoside with the observed mass of *m/z* 638.32 Da, which agreed with the fragmentation pattern of the previous report [42]. The results illustrated in a recent study suggest that, isorhamnetin-3,7-di*-O*-glucoside has potent anti-inflammatory and anticancer potential [43]. Vitexin showed its molecular ion peak at *m/z* 431.11 Da, presented fragmentation ions at *m/z* 310.61 Da, and 340.70 Da, which is related to the loss of *C*-hexosyl units attached to the aglycon unit [44]. Vitexin induced G2/M-phase arrest and cellular death in LN-18 (human glioma cells) via the Akt/mTOR signalling pathway [45]. Isovitexin which possess antioxidant and anti-inflammatory potential showed *m/z* value of 431.22 Da, further fragmented at *m/*z 315.68 Da, 368.39 Da, and 310.61 Da, which is the characteristic fragmentation pattern of *C*-linked glycosyl groups attached to the flavones moiety [46, 47]. Iridin with the depicted mass of *m/z* 521.216 Da, showed further fragmentation into *m/z* 358.81 Da with the loss of its sugar unit [48]. Iridin addresses tumor tissue function in glioblastoma cells and inhibits intracranial tumor growth in two different in vivo mouse models [49]. Rutin showed its molecular peak at *m/z* 609.141 Da and produced fragmentation ions at *m/z* 298.05 Da, 299.05 Da, and 300.06 Da by releasing its sugar part as identified previously [50]. Rutin is a flavonol glycoside found in many edible plants has strong antioxidant properties. Studies suggest that rutin induces cell death and modulates different cellular pathways in cancerous cells [51]. Kaempferol-3-*O*-rutinoside with the *m/z* value of 593.15 Da, further fragmentated at *m/z* 284 Da, and *m/z* 283 Da due to the subsequent loss of rhamnosyl and glucosyl units from the parent ion. The observed fragmentation at *m/z* 255 Da, was due to the fragmentation of the kaempferol unit [52]. Kaempferol inhibits viability, decreases proliferation, and regulates DNA repair system in human glioma cells via non-homologous end joining repair [53].

The study identified 28 compounds, in which 3 compounds were flavonoid-*O*-glycosides, 1 flavonoid-*C*-glycoside, 2 flavone-*C*-glycosides, 2 flavanol glycosides, 1 flavanone-*O*-glycoside, 1 flavone glycoside, 3 isoflavones, 1 flavonoid, 1 flavanone, 4 triterpenoids, 3 amino acids, 1 fatty acid, 3 glycerophosphoethanolamines, 1 glycophosphoinositol, and 1 compound was trisaccharide sugar. This is the first report for the identification of flavonoids-*O*-glycosides, flavonoid-*C*-glycosides, and triterpenoids in *C. graveolens.*

### Total phenolic and flavonoid content

Secondary metabolites, such as phenolic acids, flavonoids, flavones, flavonoid glycosides, and flavone glycosides, have been identified as the compounds responsible for exhibiting free radical scavenging activities and associated health advantages [54]. Calorimetric tests, such as TPC and TFC, are often used in the evaluation of phenolic and flavonoid levels in a particular sample [25]. Using the calibration curve of gallic acid (y = 0.0039x–0.0624, R2 = 0.998) and quercetin (y = 0.0067x–0.4458, R2 = 0.928), phenolic content and flavonoid content of *C. graveolens* and its fractions were calculated as shown in Table 2.

**Table 2:** Total phenolic content and total flavonoid content of *C. graveolens* crude extract and fractions

Extract	Total phenolic content (µg GAE/mg)	Total flavonoid content (µg QE/mg)
Crude	338.7 ± 1.34^a^	177.04 ± 0.16^a^
*n*-Hexane	43.4 ± 0.36^b^	1.67 ± 1.20^b^
Chloroform	71.9 ± 1.52^b^	44.35 ± 0.18^c^
Ethyl acetate	115.4 ± 0.73^c^	54.8 ± 1.31^d^
*n*-Butanol	31.4 ± 0.42^d^	15.95 ± 0.01^e^
Aqueous	16.1 ± 0.01^d^	0.92 ± 0.26^e^

The values designated by the different letters are significantly different (Post hoc analysis followed by Bonferroni multiple comparison test, *p* < 0.05)

Solvents such as hydroalcoholic mixtures are the most suitable systems for the extraction of phenolic compounds. Water acts as a swelling agent for plant material, while alcohols lead to dehydration and collapse of plant cells, causing the breakdown of the solute-cell wall bond. Therefore, a synergistic effect is observed when water-alcohol mixtures are utilized, leading to increased flavonoid extraction [55]. As the results shows, the TPC and TFC content of the crude extract of *C. graveolens,* were determined to be 338 µg GAE/mg, and 177 µg QE/mg, respectively. Studies on the chemical structure of flavonoids reveal that their solubility is affected due to their capacity to form hydrogen bond with solvents. Polar flavonoids have affinity for solvents such as aqueous and pure alcohols, whereas moderately polar and nonpolar counterparts such as isoflavones, flavanones, flavones, and flavanols have affinity to solvents such as ethyl acetate, chloroform, dichloromethane, and diethyl ether [55]. Our study shows that the ethyl acetate fraction had good recorded values of 115.4 µg GAE/mg and 44.35 µg QE/mg, while the chloroform fraction showed moderate TPC and TFC content of 71.9 µg GAE/mg and 44.35 µg QE/mg, respectively. The fractions of *n*-hexane and *n*-butanol exhibited values of 43.4 µg GAE/mg and 1.67 µg QE/mg, and 31.4 µg GAE/mg and 15.95 µg QE/mg, respectively. The aqueous fraction had the lowest TPC and TFC values, measuring 16.1 µg GAE/mg and 0.92 µg QE/mg, respectively. TPC and TFC values are used to quantify the levels of polyphenols, phenols, flavones, flavonoids, and other antioxidant groups [54]. The investigation demonstrated that the crude extract of *C. graveolens*, together with its ethyl acetate and chloroform fractions, had a substantial presence of polyphenolic and flavonoid compounds. Phenolic substances, including flavonoids, phenolic acid, and tannins, exhibit a wide range of biological actions, such as anti-inflammatory, anti-carcinogenic, and anti-atherosclerotic properties. The observed correlation between these activities and their antioxidant potential might be attributed to the presence of polyphenolic compounds [56].

### Antioxidant assays

The assessment of antioxidant activity serves as a crucial measure for evaluating the quality of various food products, dietary supplements, and extracts derived from plants [57]. The importance of antioxidants in preserving human health and their potential in preventing and treating diseases is well-documented in multiple studies, mostly attributed to their ability to reduce oxidative stress [57]. Evaluating the in vitro antioxidant activity of an antioxidant sample using a single analytical technique is insufficient due to the diverse range of processes that an antioxidant compound or combination might exhibit in vivo [58]. The in vitro assays used in the current research are well regarded due to their expediency, simplicity, cost-effectiveness, and reproducibility as methods for evaluating the antioxidant potential of botanical extracts. Consequently, these findings may provide valuable insights into the antioxidant potential of *C. graveolens*.

According to the data shown in Table 3, the investigation of radicals scavenging of the crude extract and fractions of *C. graveolens* through the ABTS method may be ranked as follows: ethyl acetate, chloroform, *n*-butanol, aqueous, *n*-hexane, and crude extract. The IC_50_ value of the ethyl acetate fraction is 39.50 µg/mL, whereas the chloroform fraction has an IC_50_ value of 50.99 µg/mL.

**Table 3:** Antioxidant activity of *C. graveolens* and its organic fractions. Description of IC_50_ values with (95% confidence interval) in µg/mL for each sample

Extract	ABTS (IC_50_ µg/mL)	DPPH (IC_50_ µg/mL)	Metal chelation (IC_50_ µg/mL)
Crude	182.9 ± 2.26^a^	142.8 ± 2.15^a^	184.9 ± 2.26^a^
*n*-Hexane	168.5 ± 2.22^a^	190.6 ± 2.28^b^	125.1 ± 2.09^b^
Chloroform	50.99 ± 1.70^b^	44.12 ± 1.64^c^	77.20 ± 1.88^c^
Ethyl acetate	39.50 ± 1.59^b^	32.27 ± 1.50^c^	53.46 ± 1.72^c^
*n*-Butanol	89.94 ± 1.95^c^	70.79 ± 1.85^d^	129.4 ± 2.11^b^
Aqueous	115.6 ± 2.06^d^	165.6 ± 2.21^b^	141.9 ± 2.15^b^
Ascorbic acid	19.19 ± 1.28^e^	16.61 ± 1.22^e^	25.58 ± 1.40^c^

The values designated by the different letters are significantly different (Post hoc analysis followed by Bonferroni multiple comparison test, *p* < 0.05)

**Fig. 3:**
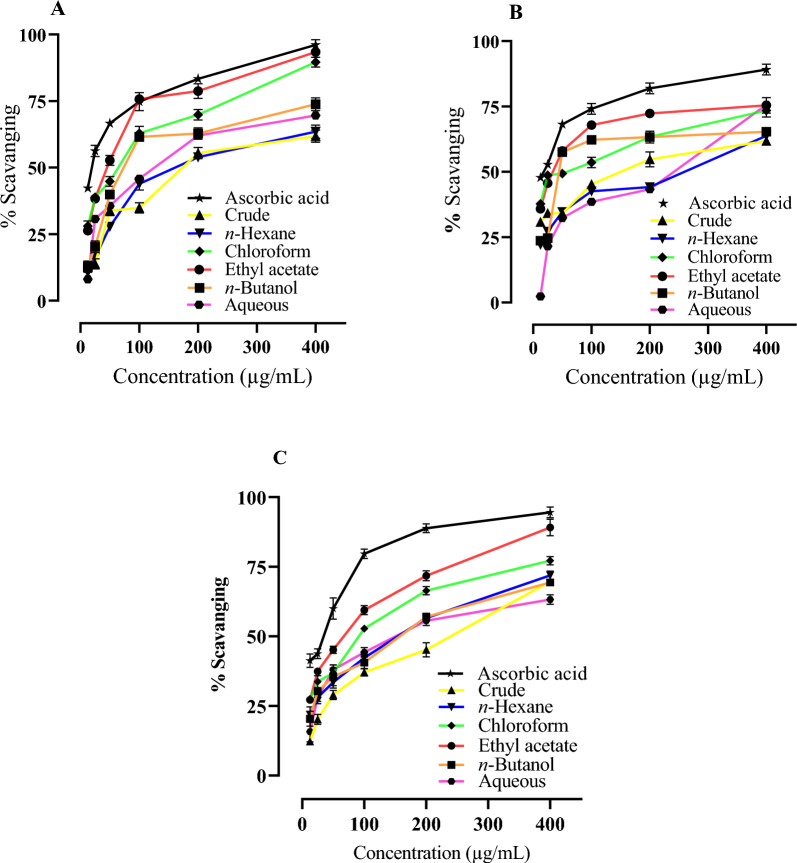
Antioxidant activity of *C. graveolens* and its organic fractions. **a** ABTS radical scavenging activity **b** DPPH radical scavenging activity **c** Metal chelation assay by ferrozine reagent. Data are presented as percentage of radical scavenging. Each value is expressed as mean ± standard deviation (*n* = 3)

The ethyl acetate fraction exhibited the maximum level of free radical scavenging activity, measuring 93.40% at a concentration of 400 µg/mL. This was closely followed by the chloroform fraction, which had a scavenged 89.60% of free radicals at the same concentration (Fig. 3A). The findings of this study indicate that ethyl acetate and chloroform fractions exhibit the highest levels of antioxidant capability. The fractions of *n*-butanol exhibited good antioxidant capacity, but the crude extract of *C. graveolens* had the lowest level of antioxidant activity.

The assessment of the antioxidant ability of extracts is often conducted using the DPPH radical scavenging activity due to its simple and robust nature [59]. The results observed for the DPPH assay indicate that the ethyl acetate fraction had the highest scavenging ability, as seen by its IC_50_ value of 32.27 µg/mL. Following this, the chloroform fraction demonstrated a scavenging capacity with an IC_50_ value of 44.12 µg/mL. The *n*-butanol fraction showed an IC_50_ value of 70.79 µg/mL, whereas the crude extract exhibited an IC_50_ value of 142.8 µg/mL. The aqueous fraction and *n*-hexane fraction exhibited a lower antioxidant potential, as seen by their IC_50_ values of 165.6 µg/mL and 190.6 µg/mL, respectively. The results shown in this assay were in accordance with the antioxidant potential observed through the ABTS activity. Figure 3B illustrates that the ethyl acetate fraction and chloroform fractions had the most significant scavenging activity, reaching a maximum of 75% at a concentration of 400 µg/mL.

Metal chelation is often considered a simple, straightforward, and commonly used method of antioxidant technique [59]. Antioxidants have been found to effectively bind metal ions due to their functional groups that perform metal binding. The biological effects of polyphenolic substances such as their antioxidant capabilities, may also change due to the interaction with Fe ions [59]. The sequence of iron metal chelation activity for *C. graveolens* and its fractions is as follows: ethyl acetate > chloroform > *n*-hexane > *n*-butanol > aqueous > crude, as shown in Table 3. The ethyl acetate fraction exhibited maximum antioxidant activity, as shown by its IC_50_ value of 53.46 µg/mL. This was followed by the chloroform fraction with an IC_50_ value of 77.20 µg/mL, the *n*-hexane fraction with an IC_50_ value of 125.1 µg/mL, the *n*-butanol fraction with an IC_50_ value of 129.4 µg/mL, the aqueous fraction with an IC_50_ value of 141.9 µg/mL, and the crude extract with an IC_50_ value of 184.9 µg/mL.

The presence of phenolic components, as revealed in Table 1, may be responsible for the extracts' considerable antioxidant ability. The findings of this study indicate that the ethyl acetate and chloroform fractions exhibit the highest antioxidant capacity. This observation is substantiated by the presence of a significant number of polyphenolic components, as well as the quantitative analytical results obtained for these fractions (Table 2). The findings of the present investigation indicate that *C. graveolens* exhibits a substantial abundance of polyphenolic chemicals, hence contributing to its antioxidative potential. According to the findings of this study, oxidative stress-related illnesses, such as cancer and other related ailments, can be effectively managed with plant extracts from *C. graveolens*.

### Anti-glioma activity

The anti-glioma activity of *C. graveolens* and its fractions were evaluated in vitro against human glioma (U-87) and human kidney (BHK-21) cell lines for cytotoxic evaluation using the MTT assay. *C. graveolens* extract and its fractions were applied to U-87 cells for 24 h, there was a gradual decrease in cell survival ratio, when compared to cells treated with DMSO (the control). According to the data shown in Table 4, it can be seen that the crude extract of *C. graveolens* revealed the maximum level of cytotoxicity, as evidenced by its IC_50_ value of 112.0 µg/mL against U-87 cell lines which can be attributed to the presence of high polyphenolic content as discussed earlier [56].

**Table 4:** IC_50_ values of *C. graveolens* and its organic fractions against U-87 cell lines

Sample name	U-87 IC_50_ (µg/mL)
Crude	112.0 ± 2.04
*n*-Hexane	167.8 ± 2.22
Chloroform	142.7 ± 2.15
Ethyl acetate	138.1 ± 1.98
*n*-Butanol	176.1 ± 3.31
Aqueous	1614 ± 3.20

**Fig. 4:**
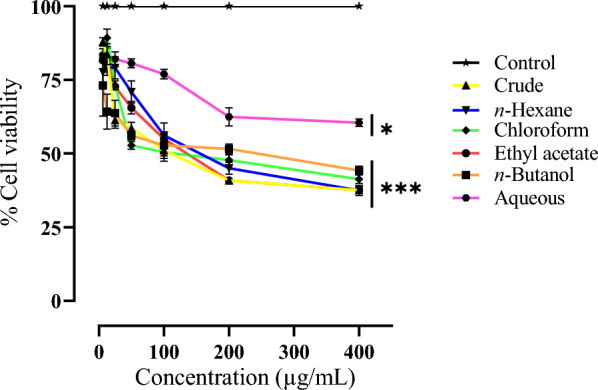
Presentation of growth inhibitory effect of *C. graveolens* extract and its fractions on U-87 cells. Results are expressed as percentage of negative. Data are means ± SD of three independent experiments. * Represent *p* ≤ 0.1, *** represent *p* ≤ 0.001

The cytotoxic effects of the ethyl acetate and chloroform fractions were found to be considerable, as shown by their IC_50_ values of 138.1 µg/mL and 142.7 µg/mL, respectively. In contrast, the *n*-hexane and *n*-butanol fractions exhibited moderate cytotoxicity, with IC_50_ values of 167.8 µg/mL and 176.1 µg/mL, respectively. The aqueous fraction exhibited the lowest cytotoxicity, as shown by its IC_50_ value of 1614 µg/mL against U-87 cell lines. The findings indicated a concentration-dependent reduction of cell viability as shown in Fig. 4. All fractions exhibited the highest percentage at a concentration of 400 µg/mL.

Plant-derived natural compounds continue to play a significant role in pharmacotherapy, particularly in the treatment of cancer and infectious disorders [60]. Polyphenols have been the subject of substantial research in the context of cancer therapy among the many phytoconstituents [61]. In a study, schaftoside has shown autophagy-induced anti-melanogenic effect in B16F1 cells [62]. Rutin has reported antiproliferative potential against different cancer cells such as lung cancer, colorectal cancer, breast cancer, and neuroblastoma [63]. Vitexin and isoquercetin have shown anticancer effects in various in vitro studies via inducing apoptosis [41, 64]. Kaempferol-3-*O*-rutinoside suppressed lung adenocarcinoma in A549 cell lines through calcium signalling pathway [65]. In another study, kaempferol-3-*O*-rutinoside suppressed ovarian cancer cells by inhibiting p-STAT3/Bcl-2 [66]. Similarly, the cell viability assay of rhoifolin revealed antiproliferative potential in pancreatic cancer by upregulating JNK while downregulating p-AKT pathway [67]. Hence, it is very much possible that the anti-glioma activity of crude extract of *C. graveolens* followed by its ethyl acetate and chloroform fractions might be due to the presence of these compounds. The findings of this study provide the basis for the traditional claim of *C. graveolens* being used in the treatment of tumors [19]. These results show that the ethyl acetate and chloroform fractions of *C. graveolens* extracts are suitable for the isolation of natural metabolites and their anticancer activities, especially in U-87 cell lines.

**Fig. 5:**
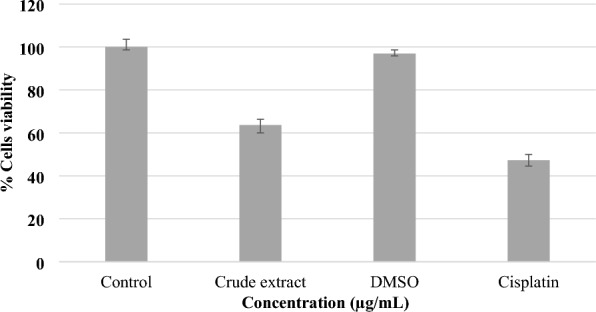
Presentation of growth inhibitory effect of *C. graveolens* extract against human kidney (BHK-21) normal cells

In a similar manner, crude extract of *C. graveolens* was assessed against human kidney (BHK-21) cell lines through MTT assay. The maximal concentration tested in this study was 600 µg/mL, as illustrated in Fig. 5. The experimental findings revealed that the extract of *C. graveolens* exhibited little toxicity toward normal cells, as seen by the data provided in Table 5.

**Table 5:** Growth inhibitory effect of *C. graveolens* against BHK-21 cell lines. Results are expressed as mean ± SD, (*n* = 3)

Sample name	% Viability ± SD	% Cytotoxicity ± SD
Control	100	0
Crude extract	63.6 ± 3.56	36.39 ± 3.56
DMSO	96.95 ± 1.67	3.05 ± 1.67
Cisplatin	47.3 ± 2.65	52.7 ± 2.65

The primary focus during the development of novel medicinal agents is to ensure safety [66]. This investigation revealed that the *C. graveolens* extract had a little cytotoxic impact on normal cells, indicating a favourable safety profile for the extract. An approach to selectively eradicate cancer cells while minimizing harm to healthy cells is crucial in order to mitigate adverse effects on bodily organs [68]. Hence, it may be concluded that the extract of *C. graveolens* is deemed to be safe for isolating elements with potential anticancer properties.

### Molecular docking studies

**Fig. 6.**
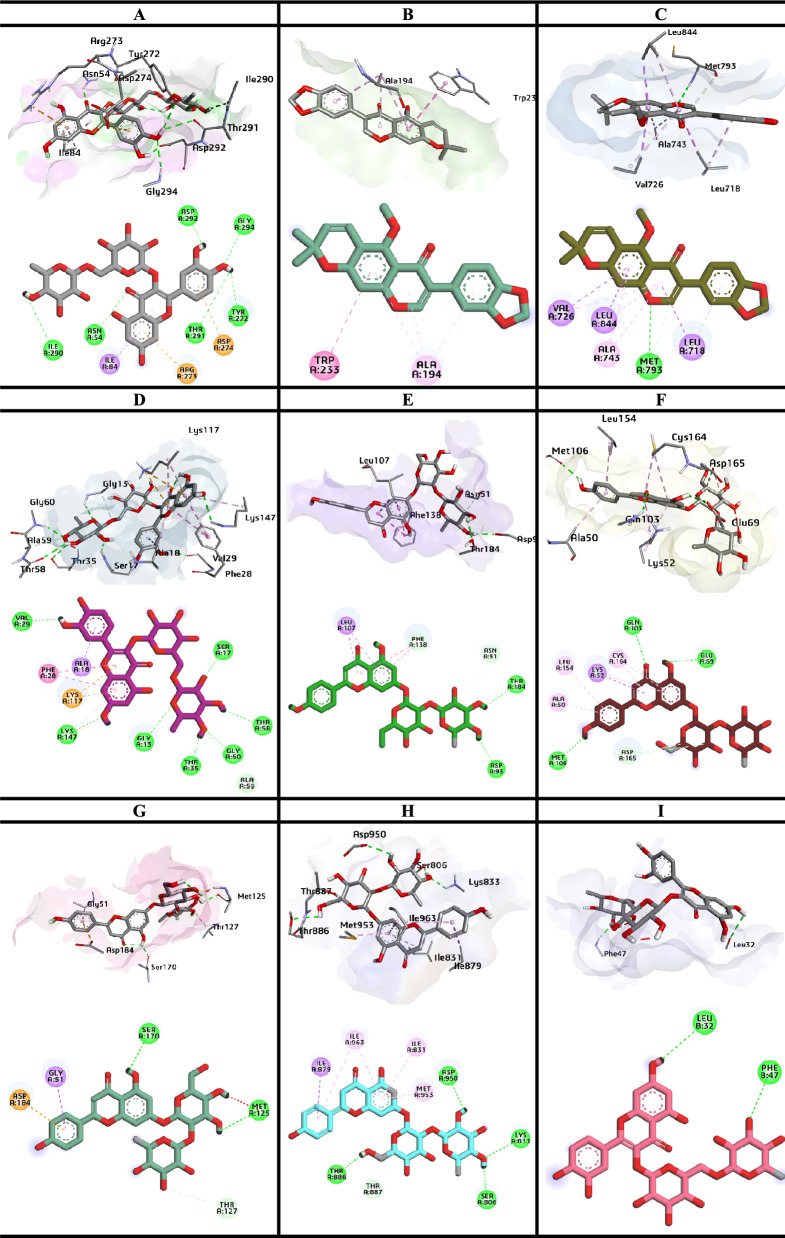
2D and 3D binding interactions of the most probable docked confirmations of the compounds; **a** Rutin **b** Methyl robustone **c** Eatoside **d** Rutin **e** Rhoifolin **f** Rhoifolin **g** Rhoifolin **h** Rhoifolin **i** Methyl robustone against targeted proteins

Molecular docking studies of the phytoconstituents present in the most potent fractions were performed i.e., ethyl acetate fraction, and chloroform fraction of *C. graveolens*. Compounds identified through the LC–ESI–MS/MS-based molecular networking analysis in the mentioned fractions were studied for the possible binding interactions and the underlying mechanisms inside the active site of the targeted gene proteins in the treatment of glioma [69]. The binding energies of all the compounds are shown in Table 6. Against the respective protein. While the compounds with highest binding energies are illustrated with 3D and 2D images in the given Fig. 6.

**Table 6:** Binding energies of the compounds with the targeted proteins (kcal/mol)

Ligand	6S9W	3AY5	7AEM	4XVR	4BQG	6RFP	6GES	7JWE	6D3O
Elatoside E	− 9.7	− 8	− 10.2	− 9.2	− 9.1	− 8.9	− 9.2	− 8.7	− 7.2
Ginsenoside Rb2	− 9.8	− 8.6	− 9.7	− 8.6	− 7.4	− 8.4	− 8.5	− 8.6	− 6.8
Ginsenoside Rb3	− 9.4	− 7.6	− 9.9	− 8.2	− 8.4	− 8.5	− 8.6	− 8.5	− 7.9
Iridin	− 10	− 6.9	− 8.8	− 8.5	− 8.5	− 8.4	− 8.6	− 8.1	− 6.9
Isovitexin	− 10.6	− 7.5	− 8.3	− 9.1	− 9.2	− 8.4	− 8.9	− 8.7	− 7.2
Prunin	− 10.3	− 7.7	− 8.9	− 10	− 9.7	− 8.7	− 8.8	− 9.4	− 7.1
Sissotrin	− 10.4	− 7.2	− 9.1	− 9.2	− 9.1	− 8.3	− 8.2	− 8.2	− 7.5
Rutin	− 11.5	− 7.3	− 9.2	− 10.8	− 10.4	− 8.5	− 9.0	− 8.8	− 7.5
Methyl robustone	− 11.1	− 9.3	− 10.2	− 8.3	− 8.6	− 8.5	− 9.1	− 9.6	− 8.0
Vitexin	− 11.1	− 7.2	− 9.1	− 9.2	− 10.5	− 7.7	− 8.6	− 9.6	− 7.1
Isoquercetin	− 9.6	− 7.5	− 8.4	− 9.3	− 9.9	− 8.0	− 8.6	− 9.6	− 7.2
Kampferol-3-O-rutinoside	− 11.3	− 7.3	− 9.2	− 10.3	− 9.9	− 8.4	− 8.7	− 9.7	− 7.1
Rhoifolin	− 11.1	7.9	− 9.4	− 9.8	− 11.1	− 9.0	− 9.2	− 10.2	− 7.4

The mechanisms of natural compounds are very complex because they have multiple potential targets and active components. Therefore, different potential targets were selected such as, AKT1, EGFR, MAPK3, HRAS, MAPK1, CCND1, HSP90AA1, and MTOR. The selected compounds from the most bioactive fractions were docked against nine targeted proteins i.e. 6S9W, 3AY5, 7AEM, 4XVR, 4BQG, 6RFP, 6GES, 7JWE, and 6D3O, respectively. The intersection targets between compounds and glioma-related genes were used to construct possible interactions. Lower binding energy of inhibitor against the targeted receptor indicates a stabler conformation i.e. the study showed the binding energies of the compounds in the range of − 7.1 to − 11.5 kcal/mol. Compounds with the lowest binding energies such as rutin, methyl robustone, and rhoifolin were further evaluated to find the binding interaction at the active pocket of the proteins. Rhoifolin showed binding energies of − 11.1, − 9.0, − 9.2 and − 10.2 kcal/mol with four targeted proteins i.e. 4BQG, 6RFP, 6GES and 7JWE respectively. The hydrogen bonding interactions with the active amino acid residues at the active pocket are as follows; 4BQG (THR184, ASP93), 6RFP (MET106, GLN103, GLU69), 6GES (SER170, MET125) and 7JWE (THR886, SER806, LYS833, ASP950). Methyl Robustone with the binding energies of − 9.3, − 10.2 and − 8.0 kcal/mol against three targeted proteins i.e. 3AY5, 7AEM, and 6D3O showed Van der Waal’s interactions with the TRP233, ALA194 and hydrogen bonding interaction with MET793, LEU32, PHE47. Similarly, rutin showed the lowest binding energies of − 11.5 and − 10.8 kcal/mol against two targeted proteins i.e. 6S9W and 4XVR, while its hydrogen bonding interactions with the active amino acid residues are ASP292, GLY294, TYR272, THR291, ASN54, ILE290 and SER17, THR35,58, GLY13, 60, LYS147, and VAL29, respectively. Similarly, Van der Waal’s interactions such as π-alkyl, π-π, π -sigma, and π-cation interactions were also observed in among the targeted compounds and the active pockets of targeted proteins. Among the potential targets AKT1, MTOR, CCND1, and EGFR pathways are closely associated with angiogenesis, autophagy, and apoptosis in glioma [70–72]. EGFR, particularly the Akt and MAPK signalling pathways are associated with the inhibitory effects on downstream signal molecules [73, 74]. This study shows the mechanisms of compounds present in the bioactive fractions of *C. graveolens* through docking by constructing a network in between compound-target-pathway. The findings of this study are broad, hence, in vivo studies are suggested for verification.

## Conclusion

In this study, a local plant, *C. graveolens*, was comprehensively investigated for its metabolite profiling by using LC–MS/MS-based molecular networking. A total of 714 nodes were annotated, which were divided into different compound classes. The results showed the presence of a variety of phytochemicals present in *C. graveolens* crude extract and its fractions with the identification of twenty-eight different compounds. The observed contents consisted of flavonoid-*O*-glycosides (3), flavone-*C*-glycosides (2), isoflavones (3), flavanol glycosides (2), flavonoid-*C*-glycoside (1), flavanone-*O*-glycoside (1), flavone glycoside (1), flavonoid (1), flavanone (1), triterpenoids (4), amino acids (3), fatty acid (1), glycerophosphoethanolamines (3), glycophosphoinositol (1), and trisaccharide sugar (1) were tentatively identified. The study has successfully investigated and established the total phenolic and total flavonoid content along with the antioxidant capacity of *C. graveolens* crude extract and its fractions. In order to address the increasing demand for new therapeutic agents, it is imperative to explore novel sources of anti-glioma agents. Consequently, we have investigated the anti-glioma properties of the crude extract and fractions derived from *C. graveolens* based on its uses in traditional medicines. The results of the in vitro anti-glioma investigation indicated that the crude extract, ethyl acetate fraction, and chloroform fraction of *C. graveolens* exhibited significant antiproliferative effects against U-87 cells, a human glioma cancer. Furthermore, the in silico studies support the in vitro results and justify the inhibitory potential of the compounds. The observed phenomenon may be ascribed to the higher concentration of polyphenolic compounds present in the plant. In order to assess the safety profile of *C. graveolens*, an investigation was conducted on its crude extract using (BHK-21) human normal kidney cells. Our findings indicated that the plant contains potential anti-glioma constituents that are deemed to be suitably safe. Given the diverse range of metabolites and the biological studies conducted on *C. graveolens*, it is suggested to further explore and isolate new anticancer agents from this plant.

## Data Availability

No datasets were generated or analysed during the current study.
